# Shaping and Focusing Magnetic Field in the Human Body: State-of-the Art and Promising Technologies

**DOI:** 10.3390/s22145132

**Published:** 2022-07-08

**Authors:** Sabrina Rotundo, Danilo Brizi, Alessandra Flori, Giulio Giovannetti, Luca Menichetti, Agostino Monorchio

**Affiliations:** 1 Department of Information Engineering, University of Pisa, 56122 Pisa, Italy; danilo.brizi@unipi.it (D.B.); agostino.monorchio@unipi.it (A.M.); 2 Fondazione CNR-Regione Toscana G. Monasterio, 56124 Pisa, Italy; alessandra.flori@ftgm.it; 3 CNR Institute of Clinical Physiology, 56124 Pisa, Italy; giovannetti@ifc.cnr.it (G.G.); menichetti@ifc.cnr.it (L.M.)

**Keywords:** magnetic field, focusing, magnetic hyperthermia, transcranial magnetic stimulation, metamaterials, metasurfaces, biomedical applications

## Abstract

In recent years, the usage of radio frequency magnetic fields for biomedical applications has increased exponentially. Several diagnostic and therapeutic methodologies exploit this physical entity such as, for instance, magnetic resonance imaging, hyperthermia with magnetic nanoparticles and transcranial magnetic stimulation. Within this framework, the magnetic field focusing and shaping, at different depths inside the tissue, emerges as one of the most important challenges from a technological point of view, since it is highly desirable for improving the effectiveness of clinical methodologies. In this review paper, we will first report some of the biomedical practices employing radio frequency magnetic fields, that appear most promising in clinical settings, explaining the underneath physical principles and operative procedures. Specifically, we direct the interest toward hyperthermia with magnetic nanoparticles and transcranial magnetic stimulation, together with a brief mention of magnetic resonance imaging. Additionally, we deeply review the technological solutions that have appeared so far in the literature to shape and control the radio frequency magnetic field distribution within biological tissues, highlighting human applications. In particular, volume and surface coils, together with the recent raise of metamaterials and metasurfaces will be reported. The present review manuscript can be useful to fill the actual gap in the literature and to serve as a guide for the physicians and engineers working in these fields.

## 1. Introduction

Recently, magnetic fields have been extensively used in various bioengineering and biomedical applications, both for diagnosis and therapy. In more detail, one of the most relevant advantages, over the current technologies, is the lower invasiveness on the patients, in particular in terms of non-ionizing radiations. In addition, due to the non-magnetic nature of human tissues, the tissue-air interface is not a barrier to the magnetic field, which penetrates without attenuation, unlike its electric field counterpart. Several diagnostic and therapeutic methodologies exploit this physical entity such as, for instance, Magnetic Resonance Imaging (MRI), hyperthermia and thermoablation with magnetic nanoparticles (also called Magnetic Fluid Hyperthermia, MFH) and Transcranial Magnetic Stimulation (TMS). In these biomedical applications, the Radio Frequency (RF) desirable magnetic field distribution plays a relevant role to improve the procedure effectiveness and its distribution is correlated to the purpose of the specific technologies.

For example, in MRI, the magnetic field is used for the human body imaging of several pathologies’ diagnoses in different anatomical districts. In this case, to excite a bigger anatomic area of the investigated tissue, and so to increase the field of view (FOV), it is necessary to produce a homogeneous magnetic field. Instead, in MFH, the magnetic field is used to induce tumor tissue heating for cancer therapy, by exploiting the temperature-damaging effect on cancer cells. In particular, in this application, the magnetic field has to be focused to perform a local treatment to preserve the surrounding tissue. Similarly, in TMS, due to the brain complexity, it would be desirable to focus the induced electric field inside the investigated tissue to improve the procedure’s effectiveness and accuracy, and, consequently, reduce the invasiveness of the procedure on the patients.

Due to the social impact of the aforementioned techniques, the requirement to generate the desired magnetic distribution is a great challenge for the scientific community.

In fact, the difficulty to produce a particular magnetic distribution, especially at relatively low frequencies, is a consequence of the magnetic field’s nature (affected by diffraction and attenuation phenomena). Furthermore, according to Biot–Savart’s law, as the distance from the source increases, the magnetic field beam diverges and the field amplitude decreases, at the same time.

**Figure 1:**
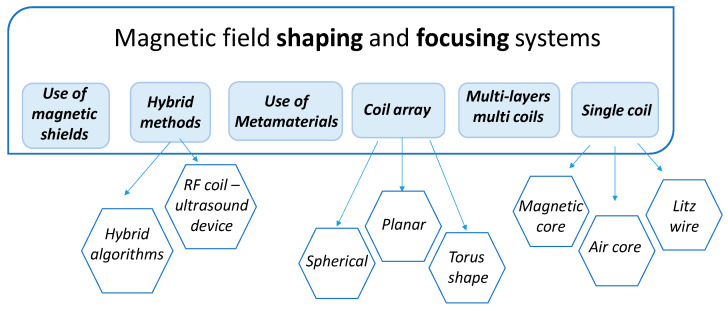
Scheme of the radiating solutions for magnetic field shaping classified by the adopted approach.

For these reasons, the scientific community’s research has been directed toward the study of different technologies able to optimize and control this aspect. In this paper, we report the most important applications in which magnetic field shaping has a fundamental role, describing the different innovative radiating solutions proposed in the open literature (Figure 1) and discussing the state-of-the-art and future emerging technologies.

The manuscript is organized as follows: Section 2 is devoted to introducing some important biomedical applications that exploit RF magnetic fields; in Section 3 we report the most meaningful hardware configurations proposed in the state of art for the different applications, with discussions about performance and applicability. Finally, Conclusion follows.

## 2. Biomedical Applications Exploiting Magnetic Field

As introduced, radiofrequency and microwave magnetic fields are widely applied in biomedical applications; therefore, several radiating configurations have been developed in the literature to shape and focus the magnetic field in order to optimize the treatment or diagnosis effectiveness. Nevertheless, it is most important to introduce the physical principles and hardware requirements of some of the most significant clinical applications before reviewing the developed radiating elements.

### 2.1. Hyperthermia and Thermoablation

Local hyperthermia is a cancer treatment technique that harnesses the heat produced by electromagnetic or acoustic fields (for instance through radio waves, microwaves, or ultrasound) generated by inductor devices located at the surface of superficial cancer or implanted inside small tumor regions [1].

**Figure 2:**
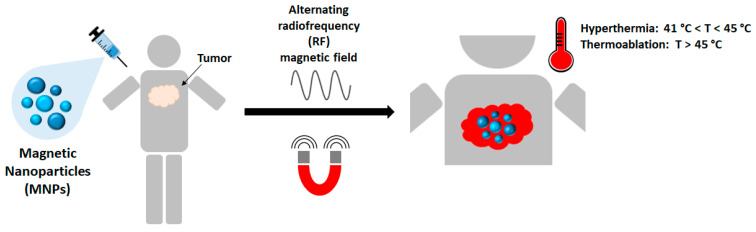
Sketch of hyperthermia and thermoablation procedures. The heating medium (i.e., magnetic fluid) is injected in the targeted region; by exciting such fluid with an external RF magnetic field, it is feasible to significantly increase the temperature in a local manner.

In particular, MFH [2] consists in inducing a ferrofluid temperature increase, composed of magnetic particles (such as iron-oxide nanoparticles) dispersed in an aqueous medium, excited under an alternating magnetic field (AMF) [3]; its application has been proposed since the late 1950′s [4] (Figure 2).

In MFH, the magnetic energy conversion to heat occurs through dissipation processes induced by the combination of different mechanisms, including hysteresis, Brown relaxation, and Néel relaxation. In particular, the AMF frequency, the magnetic particle size and the magnetic field strength determines which mechanism predominates.

Furthermore, the multi-domain magnetic nanoparticles heating (MNPs) (sizes larger than approximately 40 nm depending on the magnetic field parameters), is mainly due to hysteresis losses [5]. Conversely, for single domain particles with diameters lower than 40 nm, so-called super-paramagnetic iron-oxide nanoparticles (SPION), Brownian and Néel relaxations are the main mechanisms of heat conversion for AMF frequency above 100 KHz. Finally, for smaller iron-oxide nanoparticles (~10 nm), Néel relaxation becomes the dominant mechanism at the AMF frequencies typical of hyperthermia studies [6,7].

In brief, in the presence of a time-varying magnetic field, the MNPs realign their magnetic moments to the applied field, characterized by the Brownian and Néel relaxation time constants [8]. Brown relaxation governs the reorientation of the magnetic particle itself in a fluid, resulting in friction between the particle and the fluid. Instead, Néel relaxation derives from the magnetic moment reorientation within the particles, in opposition to the anisotropy energy that tends to orient the magnetic domain towards a given direction with respect to the crystal lattice [6,9]. As the magnetic field excitation frequency increases, the nanoparticles’ magnetic moments accumulate a phase lag with respect to the applied field. The resulting power dissipation associated with this misalignment leads to an increase in the MNPs bulk temperature and their surroundings [8].

In magnetic hyperthermia experiments, the target tissue temperature is mildly increased between 41 °C and 45 °C, for cancer-treating purposes, without inducing direct ablation. Indeed, tumor cells are more temperature-sensitive than normal cells, thus moderate hyperthermia treatment for a duration of 30–60 min induces tumor cells apoptosis and increases their response to radio and chemotherapy [10]. Moreover, the degenerated cells’ multiplication rate is reduced under increased temperatures [11]. Compared to other cancer treatment techniques, MFH provides several advantages, such as the lack of ionizing radiation and of penetration depth limitations (which means that it can be used to treat tumors located anywhere in the body). In addition, MFH is noninvasive and elicits a temperature increase highly localized to MNPs, at the same time minimizing background tissue heating [12].

Following the first application for inductive heating of dogs’ lymph nodes [4], several phantom studies and animal experiments have been so far performed to investigate the mechanisms and the effectiveness of this technology [7,9,13]. To provide an overview of MFH in vitro and in vivo applications is out of the scope of this review, however, it is worth mentioning that the MFH technique proved more recently to be effective in clinics [12]. In particular, the NanoTherm^®^ therapy showed promising clinical results in glioblastoma multiforme treatment. In 2010, this therapy received approval from the European Medicines Agency (EMA) for cancer treatment and had an Investigational Device Exemption (IDE) approved by the Food and Drug Administration (FDA) for prostate cancer treatment [12]. Nevertheless, the MFH introduction into standard clinical practice is not yet a foregone conclusion: real-time and accurate monitoring of temperature increase at the tumor site, together with more realistic computational modeling for better treatment planning, are crucial needs in the current state of the art [5,14].

Many research papers published in the field of magnetic hyperthermia focus on the design, synthesis and characterization of suitable MNPs [13,15,16,17]. For effective hyperthermia applications, the injected MNPs should be biocompatible, biodegradable, with good stability; moreover, they should be successfully delivered to the tumor cells and provide good heating efficiency (which means that they should be able to provide large heat generation even with small amounts) [1]. Their physicochemical properties can be tailored to improve their selectivity towards the target tissue, for instance by modulating their size or composition or through proper surface functionalization [13]. In more sophisticated applications, the MNPs can be engineered for drug encapsulation and the application of MFH can trigger controlled drug release [12]. Moreover, MNPs can be used as MRI contrast agents for theranostic studies [13]. A crucial aspect is the MNPs administration for the MFH treatment effectiveness. In particular, MNPs intratumoral administration, able to achieve a high local MNPs concentration, has been widely explored not only in experimental studies in animal models and in humans but also to investigate temperature increase effects at the cellular level in isolated cells experiments. Intravenous MNPs administration has been also investigated: in these studies, the hyperthermia treatment effectiveness could be hampered by uncontrolled and limited MNPs accumulation at the tumor site, reduced MNPs circulation time and MNPs sequestration by the immune system [5,14].

Experimentally, the MNPs heating power value can be evaluated by measuring colloidal solution temperature rise placed in AMFs. However, given that the heat generated by the MNPs during one cycle is equal to their hysteresis loop area, an alternative method consists of directly measuring the hysteresis loop, which is more informative and much faster than temperature measurements [18]. Alternatively, the MNPs potential evaluation of heating power value can be performed through measurements of their specific absorption rate (SAR), defined as the absorbed energy per unit of nanoparticle mass. SAR is dependent on the magnetic field frequency and intensity and on nanoparticle size, shape, material, agglomeration rate and dispersion media. The required magnetic field for performing SAR measurements is generated by electromagnetic applicators, whose architecture should be designed for generating a controllable highly homogeneous magnetic field in a defined volume at different frequencies and intensities [19].

As mentioned previously, one of the magnetic hyperthermia’s main advantages is that in principle it provides local heat deposition and does not damage healthy cells surrounding the tumor; in fact, the observed temperature increase occurs only within the volume containing the MNPs and the closely adjacent tissues [11].

However, heat localization to the tumor region is critical, since the MNPs have the tendency to migrate from the tumor site towards healthy tissues during MFH experiments. Moreover, some organs, such as kidneys and liver, are devoted to accomplishing the final depletion of exogeneous substances and, for this reason, they always contain a certain not-desired MNPs concentration.

Injected MNPs localization can be improved, for instance, through the addition of a static magnetic field (SMF): in particular, the SMF sources are positioned so that the SMF vectors bend each other, and a field-free region (FFR) occurs in the workspace. In this way, while MNPs in the SMF are restricted or completely blocked, MNPs that remain in the FFR can oscillate freely under the influence of AMF [20].

As reported in [21], an alternative method consists of using “superlocalization” procedures, based on the combination of the applied oscillating magnetic field with a static field gradient: the larger the static field the smaller the dissipation and, consequently, the temperature increase is observed mainly where the static field vanishes. The authors also investigated how thermal effects impact the superlocalization effect of MNPs hyperthermia for oscillating and rotating applied fields. In the low-frequency range useful for magnetic hyperthermia, the oscillating applied field resulted in SAR two times larger than the rotating one with identical superlocalization ability, which means that by comparing the rotating and oscillating applied fields at the same frequency, the latter provides a better heating efficiency in the frequency range suitable for medical applications [21].

Few other papers in the literature concerning a rotating magnetic field application as an effective alternative to more commonly used AMF in hyperthermia experiments have been proposed [11,22,23,24,25].

A procedure and a technological setup similar to MFH can be used for thermoablation, a cancer treatment technique in which tissues are heated above 50–60 °C, for a duration of 4–6 min. Compared to hyperthermia, this technique aims to induce extensive and direct tumor cells’ necrosis [26]. In fact, magnetic thermoablation applications, by combining nanotechnology and thermal therapy, employ MNPs as an internal source for selective heat generation after exposure of the tumor to an AMF [27]. Applications of thermoablation using MNPs for the treatment of breast [28], prostate [29], glioblastoma [30], and kidney cancer [31] have been reported in the literature.

Alternative thermoablation methods, so-called thermoablation by means of Radiofrequency (RFA), have been also proposed. In particular, in this application the inductive heating is obtained through an AMF from outside the body, closing the circuit by placing a small implant (i.e., an electrode) within the body. In particular, bipolar RFA is based on the use of two electrodes between which an RF current alternates, while a probe is inserted into the body until it is in contact with the target tissue, whose electrical resistance creates a circuit between the two electrodes. When the alternating current is introduced into the tissue, the ions’ agitation causes tissue coagulation and friction, resulting in cell death. Instead, monopolar RFA consists of inserting a single needle or catheter electrode into the body and inducing the current by using a grounding pad on the skin. RFA has been employed for treating lesions in the kidney, liver, bone, lung, breast, pancreas and prostate [32].

Another possibility for thermal ablation for treating cancer is Microwave ablation (MWA). In this technique, water molecules in the body vibrate under electromagnetic waves in the microwave energy spectrum (300 MHz to 300 GHz) and the frictional heat, produced by this vibration, causes a temperature rise in living tissue [33].

In both RFA and MFA techniques, one of the most relevant disadvantages is the direct electrode insertion, to induce heating, in the body tissue itself, causing pain and distress in patients.

### 2.2. Transcranial Magnetic Stimulation

Transcranial Magnetic Stimulation (TMS) is a promising non-invasive, electromagnetic technique for brain stimulation. Due to its theoretical simplicity and effectiveness, it is increasingly adopted and studied, by the scientific and medical community, especially for neuronal tissue excitation. Indeed, its potentialities in terms of clinical treatment or for studying functional activities, such as Parkinson’s disease and neuronal and psychiatric disorders such as depression, are particularly significant. When the technique is employed for depression treatments, often repetitive magnetic pulses are delivered (repetitive TMS or rTMS). Clearly, TMS is an alternative to traditional neuronal treatment with substantial advantages with respect to electroconvulsive therapy (ECT) and electrical stimulation, far more invasive on the patient.

This innovative technique is, in fact, painless, less expensive, faster, and, at least in some cases, more effective than conventional electrical stimulation, as in transcranial electric stimulation (TES), which generally requires active electrodes to be implanted inside the tissue. One of the most important differences between these two methods is the density level of the induced currents. In particular, because of the low skull conductivity, TES requires the application of a large electric potential between the electrodes, inducing an excessive current density in the human scalp. Due to the different generating induced currents physics, in TMS the produced maximum current density in the skull is much lower than in TES, allowing pain reduction for the patients. Moreover, TMS treatment can be performed directly on an awake patient, eliminating the need for anesthesia, strictly necessary in the other methodologies, thus becoming safer.

In addition, it is known that the skin is one of the parts of the human body in which the nerve endings devoted to detecting pain are located. Thanks to magnetic stimulation, it is possible to induce a painless treatment because the induced currents do not pass through the skin, and, consequently, it does not stimulate the nerve endings. Moreover, TMS induces relatively diffused currents and consequently, there are no high and dangerous peak currents which, by contrast, occur with electrical stimulation.

In a general arrangement, the TMS hardware system is located near the scalp in order to generate a magnetic field that, in turn, induces an electric field via eddy currents in the conductive brain tissue. When the spatial gradient of the induced electric field aligns with a nerve fiber, an action potential is generated. More specifically, the physical concept behind this method can be observed in magnetic induction law. It is well described by Maxwell’s first equation in the time domain:

(1) $$\oint \vec{e}\cdot \vec{l_{l}}\cdot d_{l}=-\frac{d}{dt}∯\vec{b}\cdot \vec{l_{n}}\cdot d_{S}$$

where the integral at the first member represents the induced electromotive force and the second member is the flux of the magnetic vector through an assigned surface *S*. This equation, known as Faraday–Lenz law, expresses the correlation between the variation in time of the magnetic flux concatenated within a closed path and the electromotive force induced on it. In particular, the electromotive force determines eddy currents that will circulate through the tissue in a plane perpendicular to the inducing magnetic field flux, so as to generate an induction magnetic field that will oppose the increase in the intensity of the inducing field strength itself. Therefore, it is possible to induce an electric field stimulation in a specific region inside the tissue by generating an appropriate magnetic field distribution, in a contactless way (i.e., without the use of implanted electrodes).

Given the clinical requirements to stimulate targeted and definite brain regions, TMS applicators have to produce large electric fields that are, at the same time, sharply focused on the required treatment region deep in the brain. This aspect is a substantial challenge since TMS coils typically generate rather diffused fields that are rapidly decaying with distance from the scalp; these fields often excite tissue well outside of the target region and/or fail to reach it altogether. For this reason, TMS coils capable of producing sharply focused fields that deeply penetrate the brain have been a long-elusive research goal. In fact, the ability to excite or block brain circuits at any desired location with innovative neurostimulation techniques is critically important, not only for understanding how the brain works but also for treating neural diseases.

### 2.3. Magnetic Resonance Imaging

Beyond the above-mentioned treatment methodologies, MRI is one of the most important non-invasive medical imaging techniques for the diagnosis and follow-up of diseases affecting different tissues and organs. For image production, MRI employs a strong static magnetic field associated with gradients and RF pulses.

The RF field is generated by transmitting coils and it is used to excite the proton atoms within the biological matter. After the high-power exciting RF pulse, the re-emitted signal is picked-up by receiving coils; both exciting and receiving coils are carefully adapted to the specific application and to the human body portion dimension. In particular, while the transmit coil has to produce a highly homogeneous magnetic field in the desired field of view (FOV), the receive coil has to maximize signal detection while minimizing the noise [34].

According to their shapes, MR coils can be categorized into volume, surface and phased-array coils. The volume coils group is constituted mainly by Helmholtz coils, solenoids and birdcage coils, which are often employed both as transmit and receive coils due to their ability on producing a uniform magnetic field in a large region surrounding the human body portion. Instead, surface coils are constituted by loops of various shapes (circular, square and elliptical) and are much smaller than the volume coils. They usually have a higher Signal-To-Noise Ratio (SNR) but relatively poor field homogeneity and, thus, are mainly used as receiving coils [35]. Finally, phased-array coils, constituted by different circular or rectangular loops, allow to achieve a good SNR, typical of surface coils, with a large sensitivity region, usually provided by volume coils [36]. The coil magnetic field pattern estimation is an important process that has to be taken into account in the coil design: such analysis can be performed by using two different approaches. The first one is based on magnetostatic theory, which implies the nearly static field assumption, valid for coils size much lower than the wavelength [37]; for coils that are not small compared to the wavelength, the interaction between RF field and the human body is relevant and magnetic field calculation can be performed with numerical simulation based on Maxwell’s equations. Such numerical methods include the Finite-Difference Time-Domain (FDTD), the Finite Element Methods (FEM) and the Method of Moments (MoM) [38].

Recent advances in RF technology have permitted the design of different geometry coils able to optimize the magnetic field pattern in a defined FOV jointly with sample-coil filling factor maximization. For example, a flexible form-fitting phased-array coil designed for being conformed to the anatomy of different sizes or shapes can significantly reduce the spatial distance between the coil and the human body portion, thus maximizing the image SNR [39]. Zamarayeva et al. [40] proposed a method employing spray deposition of silver nanoparticle inks and dielectric materials on 3D printed substrates for additive and rapid manufacturing of 3D patient-specific coils which ensure the perfect fit to the body parts with complex geometries such as the neck.

### 2.4. Critical Analysis and Comparison

As reported, several biomedical applications, both therapeutical and diagnostic, require a careful magnetic field distribution design in order to achieve efficacy and ensure patients’ safety. As a matter of fact, each of the three investigated techniques, namely magnetic hyperthermia and ablation, transcranial magnetic stimulation and magnetic resonance imaging, has its own specific requisites and needs in terms of field distribution.

In particular, magnetic hyperthermia requires a magnetic field oscillating at the correct frequency to optimize the employed magnetic nanoparticles’ response. Although the heating can be localized by directly injecting the magnetic fluid into the targeted region, nevertheless the magnetic field focalization is a highly desirable requirement to have a punctual treatment. Indeed, due to the tissues’ depletive function, the nanoparticles tend to diffuse from the targeted region and locate in surrounding organs. Instead, field focusing and shaping are of utmost importance in ablation since the required power levels are significantly higher, and serious damages to healthy tissues can happen if the RF or MW radiation is not spatially confined.

This latter aspect is also extremely important in Transcranial Magnetic Stimulation because the treatment must excite a highly localized brain region to obtain the desired clinical output. Here, the potential drawbacks of non-specific irradiation can be dramatic, therefore a lot of effort in the literature has been carried out to find coils configurations able to cope with this issue.

Finally, Magnetic Resonance Imaging can be enhanced in terms of performance by illuminating the largest Field of View with an RF magnetic field as homogeneous as possible. Therefore, besides traditional solutions based on surface and volume coils, research activity has been particularly conducted to couple RF coils with metamaterials and metasurfaces. Indeed, their capabilities to shape and control the field distribution make them ideal candidates to face and address the shimming requirements in MRI.

In the next section, the most important and significant radiating configurations able to manipulate the magnetic field that have appeared so far in the literature will be presented, together with a careful analysis and discussion.

## 3. Technical Approaches for Magnetic Field Shaping and Focusing

### 3.1. Applicators for Magnetic Hyperthermia

From a technical point of view, it is very difficult to focus the magnetic field (oscillating at hundreds of kHz) on a delimited area in magnetic hyperthermia applications, due to its huge wavelength [41]. On the one hand, the lack of RF magnetic field focusing could cause a great exposure for healthy tissues; on the other hand, the AMF used to heat the MNPs should meet safety conditions with limited frequency and amplitude ranges [1].

Therefore, suitable devices designed for AMF generation emerge as fundamental components for efficient heat deposition and successful hyperthermia applications. There are several designs of magnetic field generators used in magnetic hyperthermia experimental studies and most of them are the same employed in MRI applications as volume or surface RF coils. Custom-made and commercial setups always include one of the following components: single or multilayer solenoid, flat coil (pancake, circular, rectangular), Helmholtz or birdcage coil. Each configuration provides advantages and disadvantages in terms of magnetic field homogeneity and amplitude, with respect to specific MFH experimental applications [11,42,43,44,45,46].

In fact, apart from the technical properties, such as current demand and reactive power, the choice of the best inductor design is strictly dependent on the specific experimental conditions and in particular on the experimental model (cell culture, small or large animal model, human body) as well as on tumor location and dimensions.

**Figure 3:**
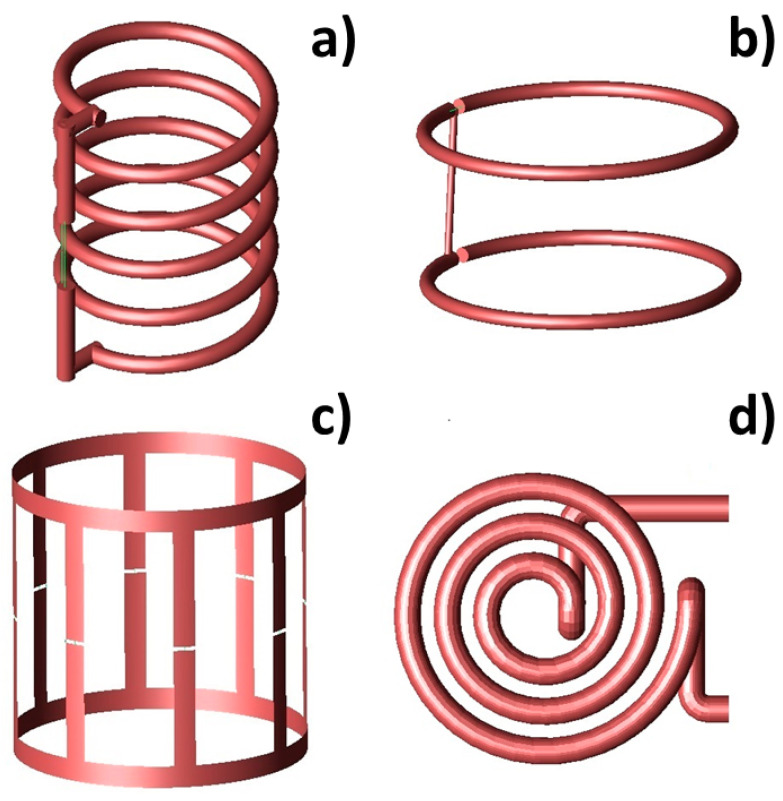
Sketch of the typical coil configurations employed in hyperthermia experiments: a solenoid (**a**), Helmholtz configuration (**b**), birdcage (**c**) and pancake (**d**) coils.

The inductor configurations currently employed for local magnetic hyperthermia experiments could be divided into two main groups: volume and surface inductors (see Figure 3).

#### 3.1.1. Volume Inductors

**Figure 4:**
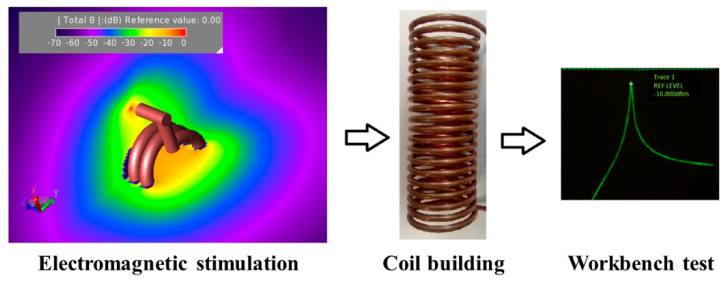
Different steps of inductors development for hyperthermia applications. Numerical design is followed by fabrication and workbenching tests.

As regards volume inductor configurations, their scope is to illuminate large specimens in the most homogeneous way possible. In this case, the magnetic field uniformity is considered an essential condition for more effective treatment in magnetic hyperthermia applications. Therefore, an optimization procedure for the magnetic field source design has to be taken into account in order to provide a control for the magnetic field uniformity and hence a uniform therapeutic temperature in wide-body regions. Nonetheless, at the same time, the capability to adapt the inductor shape and currents to the patient’s size and region to be treated should be guaranteed (Figure 4).

Among volume inductors, a favored solution is solenoids. The solenoid (Figure 3a) is a particularly simple and versatile design for induction heating coils, characterized by the production of large regions of relatively uniform field within the coil. In this case, the magnetic field strength is directly proportional to the number of coil turns and the applied current, while the magnetic field uniformity is strongly determined by the length of the solenoid [47]. Due to the ease of fabrication and high versatility, inductor configurations based on solenoids have been successfully used in a number of hyperthermia studies [6,8,19,42,48,49]. For example, in Brizi et al. [50], a solenoid was employed for acquiring experimental data of SAR measurements. In particular, an Eppendorf vial was exposed to an RF magnetic field by placing it within the inner volume of the solenoid. In this way, the exposition was extremely uniform within the entire vial. Such setup was also replicated with electromagnetic simulations performed with the commercial software Feko (Feko suite, Altair, Troy, MI, USA), based on MoM, in which the solenoid was designed through a CAD modeling with similar electromagnetic properties to the ones experimentally employed. Di Barba et al. [51] proposed an automated optimization procedure for the design of an air-cored solenoid system, robust against small variations of the coil parameters and characterized by an adaptable geometry, aimed at providing the maximum homogeneity of both magnetic field and tissue heating in the region of interest. A finite-element analysis (FEA) commercial code was employed for electromagnetic simulation, in which the tumor was modeled by a sphere inserted in a spherical volume representing the liver which, in turn, was embedded in a cylinder with an elliptical cross-section, representing the human body.

The solenoid was also proposed as an optimal inductor design for generating a homogeneous magnetic field in cell culture experiments. For example, Bertani et al. [52] presented the optimization of the magnetic field phases in a device for heating an NP magnetic fluid, previously injected inside cells cultured in a Petri dish, with the purpose to apply the same intensity of magnetic field on the cell surface and to obtain uniform heating. Blanco et al. [45] present the assessment of human melanoma cell death pathways in response to magnetic hyperthermia, with the goal to develop a real-time in situ molecular tracking system. This was achieved by a design of a dedicated instrument for the magnetic field generation in conjunction with a fluorescent/optical live-cell imaging microscope, and by developing an imaging protocol able to deliver information on cell death pathways. The controlled time-varying magnetic field was supplied by a six-turn water-cooled solenoid coil able to produce a magnetic field with near-uniform (within ±6%) intensity over a cylindrical volume of 16 mm height and 20 mm diameter at the solenoid center. A similar configuration is also described in the paper by Subramanian et al. [53] in which the RF coil could fit inside an incubation chamber, and be mounted onto a microscope for real-time cell studies.

Despite solenoids have the property of generating high peak-amplitude fields with limited stray fields extending outside the coil, they only provide a homogeneous magnetic field over a limited volume within the coil and with nonuniform 3D field patterns which extend in the axial and transverse directions [54]. In particular, a significantly inhomogeneous field distribution arises in the proximity of both coils’ ends, which has been reduced with several technological expedients. For instance, Bordelon et al. [55] employed a two-dimensional finite element analysis (2D-FEA) simulation software for designing a modified solenoid with high homogeneous field uniformity within a volume of interest for evaluating MNPs samples and for treating small animal models of human cancer. The coil had wide planar turns and two magnetic flux concentrators on both ends to significantly improve the homogeneity of the generated field. In particular, the addition of the magnetic field concentrator rings provided a 5% increase in the magnetic field amplitude and a 27% increase in uniformity with respect to the simple solenoid.

In the paper by Tang et al. [47], the authors optimized the magnetic field uniformity generated by a solenoid coil by introducing two correcting coils and investigated its influence on treatment temperature during MFH. The magnetic field device was composed of one main solenoid coil and two external correcting coils located at the end of the main solenoid and with a relatively short length. All coils were composed of the same material (copper) as well as the same number of coil turns per unit length (i.e., 20). Simulation results showed that the effect of the correcting coils mainly led to the presence of more homogenous magnetic field features at both ends of the solenoid, resulting in an increase of the effective treatment area, as well as in an enhanced thermotherapy effect even if at the expenses of a slightly more complex system.

As a further option, geometrical modifications can also permit an increase in field homogeneity and produce significant gains in control of the field distribution inside the solenoid. In this sense, Nemkov et al. [43] proposed a solution based on wrapping the turns in the plane, in order to maintain the junctions between each turn aligned along the same path and to eliminate the circumference geometrical variation of the coil. In a second approach, he explored the addition of a ring constituted by low-reluctance material to each solenoid end, which behaves as magnetic concentrator caps on each coil. The effects of these rings were to improve the field homogeneity inside the coil and diminish the current demand and lower the reactive power.

A possible alternative to the solenoid configuration is the Helmholtz coil (Figure 3b), which is able to produce a highly uniform magnetic field strictly in the volume occupied by the sample placed at the coil center. However, such a coil requires very high voltage, current, active and reactive powers for generating large-amplitude magnetic fields (32 kA/m at 150 kHz) [43,44]. According to theory, a double-layer solenoid, with the same length and the same number of ampere-turns as Helmholtz coils, provides a much higher magnetic field intensity at the expense of a significantly reduced magnetic field homogeneity compared to the Helmholtz coil [11]. Due to its interesting features, several variations of the classical Helmholtz coil have been developed in the literature. Hadadian et al. [46] proposed the design of a Helmholtz-like coil, constituted by two solenoids with a common axis connected in series, designed for in vitro magnetic hyperthermia experiments with cell culture plates or strip. This configuration guarantees that only the MNPs in the desired well effectively sense the magnetic field. Attaluri et al. [56] designed a configuration based on a Maxwell coil, which consisted of three coils oriented on the surface of a virtual sphere, whose dimensions were suitable for large animal experiments. This configuration, designed by a 2D-FEA, generated AMF with amplitudes up to 35 kA/m (peak) in a 3000 cm^3^ cylindrical volume (15–20 cm diameter) at 150 kHz frequency. Gel phantom experiments showed a magnetic field uniformity >90% in the required FOV for this configuration.

As a further volume configuration, birdcage coils have been developed for magnetic hyperthermia, based on the traditional design coming from magnetic resonance imaging. In particular, Gresits et al. [22] presented an 8-leg lowpass birdcage coil for hyperthermia experiments and for performing SAR measurements with a non-calorimetric method (Figure 3c). The coil was characterized by its ability to generate a highly homogeneous magnetic field over a relatively large sample volume [57]. The main advantage of the birdcage coil consists in its capability to generate a magnetic field perpendicularly directed to the coil axis when driven by a single signal. Conversely, when driven by a quadrature signal, such a coil produces a circularly rotating magnetic field, in contrast to a linearly polarized magnetic field as the one produced by solenoids or surface coils [58]. Another substantial advantage of using this coil for thermotherapy is that it is widely employed in MRI, and, in fact, it could be used for irradiation straight on.

#### 3.1.2. Surface Inductors

Beyond volume radiating systems, surface inductors are also frequently adopted in magnetic hyperthermia treatment, especially when the target tissue is at a shallow depth. In fact, surface inductors generally provide a simpler structure design and circuit, at the cost of a magnetic field intensity that decreases very rapidly as the distance from the coil plane increases. Therefore, surface coils are usually not suitable for treating deeply located lesions.

Indeed, according to Faraday’s Law, an alternating magnetic field *H* at a frequency *f*, concatenating across a conductive surface area, induces eddy currents around the surface perimeter. These currents provide undesired heating of healthy tissue overlaying the tumor, thus limiting the maximum value of *H* and *f* that can be safely applied to a patient and reducing the treatment efficacy, especially in terms of required exposure time. Nevertheless, because of its smaller footprint, an optimally designed single coil can lead to the minimization of eddy currents at the patient’s surface, while maximizing such currents in the underneath tumor [44].

A further advantage of surface configuration consists in the possibility to shape this radiating system from small laboratory experiments to human body scale with full flexibility; accordingly, MFH experiments in large animals can be more easily conducted using planar rather than volume coils.

Nieskoski et al. [44] studied the magnetic field optimization produced at depth by a single coil with respect to the generated superficial eddy current heating. A comparison of a Helmholtz pair against an optimized single coil was performed by defining power ratio (PR) as the ratio between the induced heat in MNPs and the surface eddy current heating. PR computation was performed by adopting superparamagnetic magnetite NPs with an average core diameter of 15 nm and an aminosilane coating. Results showed that the single-coil maximized PR for only a single value of the target depth *d* and the optimal radius value of such coil was equal to 1.187d. For treating multiple depths with a single device, therefore, the coil has to be optimized for the largest expected value of *d*. Moreover, a direct comparison of the two coils PR was performed. For example, with a 15 cm patient radius, the single coil provided a greater PR than the Helmholtz pair at MNPs target depths <4.6 cm, while the Helmholtz pair PR overcame the optimized single-coil PR for greater depths. Such findings were independent of frequency, MNPs concentration, tissue electrical conductivity, and MNPs heating rate. To further demonstrate this aspect, Wu et al. [59] propose a 300 kHz single planar coil, wound by six turns around a hollow copper pipe, for hyperthermia experiments with MNPs in pigs. Experimental results demonstrated that the planar coil was resonating at 328 kHz, providing a maximum current of 528 A and a maximum power of 17.6 kW. The magnetic field direction measured at the coil axis was approximately perpendicular to the coil plane, while its intensity fell off rapidly with distance from the coil. Such a decrease in the magnetic field intensity resulted in reduced temperature stability of MNPs, thus confirming that surface coils are best suited for superficial treatments, while volume inductors are still the best choice for deep treatments.

In order to investigate alternative coil geometries, Nemkov et al. [43] proposed the design of a rectangular coil with a magnetic core made of Fluxtrol material. Simulations were performed with Flux 2D and Flux 3D computer simulation programs and results indicated that the rectangular coil produced required field levels and uniformity with approximately six times lower reactive power than a Helmholtz coil. Subramanian et al. [53] obtained similar results using a six-, eight-turn planar coil, fabricated with 18 standard gauge copper wire. Furthermore, the results showed that the planar coil set-up provided better field amplitude compared to a solenoid, resulting to be the best fit for experiments with cell culture plates of standard dimensions.

The single planar configuration proved to be effective also for thermoablation studies. In particular, Hilger et al. [28] described a feasibility study based on the human breast adenocarcinomas implantation into immunodeficient mice and on the magnetite particle masses intratumoral injection. Animals were then exposed to an AC magnetic field (amplitude: 6.5 kA/m, frequency: 400 kHz) by using a 9 cm diameter circular coil. The procedure provided a temperature increase between 12 °C and 73 °C at different tumor locations (tumor center and periphery). Results showed that the method permitted the generation of localized heat spots in the tumor area, underlying that the deposited heat is enough for killing tumor cells after short treatment times.

As an alternative to single planar coils, spiral “Pancake” coils (Figure 3d) are characterized by an adaptable geometry, with small dimensions that can easily fit the skin surface. However, “Pancake” coils provide smaller regions of a usable magnetic field since the strong field intensity produced immediately adjacent to the coil windings rapidly decreases with both axial distance from the coil plane and radial position in front of the coil [42]. For this reason, the planar spiral “Pancake” coil is the configuration of choice for those applications requiring a magnetic field perpendicular to the body surface. Moreover, this configuration has the advantage to provide adequate space for in vivo or phantom experiments, with respect to volume coils [46]. Brizi et al. [41] proposed a 3-turn planar spiral geometry (“pancake”) which focuses the magnetic field for a targeted and precise hyperthermic treatment at a frequency of 340 kHz, optimized for the chosen MNPs. Simulations performed with an electromagnetic tool based on the Method of Moments (Feko suite, Altair, Troy, MI, USA), permitted the design of a coil able to concentrate the magnetic field in a spot coinciding with the region included in the helix inner turn and for treatments requiring a penetration depth of few cm. Such coil could handle high magnetic field amplitude (15–20 kA/m) given the high current (few hundreds of amperes) flowing in it. Similarly, Blanco et al. [45] fabricated a pancake coil constituted by five turns of copper pipe, specifically designed for in situ operations in the live-cell-imaging microscope and was mounted directly in the microscope, 5 mm above the tissue culture dish.

Pancake coils are also adopted in more complex configurations, to enhance their advantages. In particular, the paper by Durr et al. [60] deals with the design of an AMF applicator based on a split-coil unit, to induce hyperthermia in an animal tumor model using SPIONs. The split-coil unit consisted of two flat pancake coils with variable distances between 40 and 100 mm, suitable for experiments in phantoms as well as in animals.

As described, surface coils are able to handle significantly high current values, while being extremely concentrated in terms of geometrical shape. Therefore, another point needed to be optimized for AMF inductors consists in the nature of the employed wires. Indeed, the impedance of standard copper wires increases dramatically at high frequency due to skin effects. Moreover, for multi-turn inductors, their impedance increases when a large current is flowing through them due to proximity effects. In general, these problems are solved using water-cooled hollow conductors [41,59]. In addition, Litz wires have been recently proposed as an effective alternative to minimize the conductor resistance. In fact, being made of a large number of fine insulated wire strands connected in parallel at the conductor ends, Litz wires allow to evenly share the current and maximize the surface where the current flows [61]. An example of Litz wire fabrication in this field is represented by the work of Connord et al. [18], where a setup for measuring the hysteresis loops of colloidal MNPs solutions in a frequency range of 6–56 kHz and up to 80 mT has been presented. The alternating current was produced by a function generator coupled to a voltage amplifier, while the main coil, constituted by Litz wire, was air-cooled, so no water flow was required. The use of Litz wires permitted the maintenance of the coil impedance as low as possible for moderating the heat generated inside it. Similarly, their effectiveness at reducing ohmic losses and allowing for the avoidance of cooling systems was also demonstrated by Lacroix and colleagues [62]. In this latter work, a system employing Litz wires for generating a magnetic field with an amplitude of 3.82 kA m^−1^ and frequency range of 5–500 kHz has been proposed. The system adopts an efficient electromagnet, characterized by requiring low power for producing the AMF, without the need for a cooling system.

### 3.2. Applicators for Transcranical Magnetic Stimulation

As anticipated, in Transcranial Magnetic Stimulation, the radiating system focusing capability is of utmost importance since it determines the diagnosis and treatment accuracy and efficiency. Indeed, brain stimulation effectiveness is strictly related to the possibility of precisely selecting the region to be excited. For this reason, to overcome the limits associated with the current solutions, many configurations have been proposed in the literature. Nevertheless, the developed radiating systems for TMS are always variously complex arrangements of surface coils, due to the particular biological district of interest.

A very important feature in this area consists of the opportunity to excite several punctual regions with the same instrumentation. In this sense, in [63], a multi-locus TMS system has been designed, electronically controlled and excited, to be able to adjust the induced E-field maximum location and orientation within the cortex. In particular, to induce the desired E-field in the specific cortex region, the authors developed an optimization method to calculate the needed currents in the coils. In another study [64], the same research group implemented and simulated different hardware system parameters, such as coil radius and different relative spatial positions, to study how to improve the system magnetic field focalization. In particular, the results showed that the focusing performance can be improved by carefully optimizing the distance between the coils constituting the system.

Liu C. et al. [65] proposed another solution, in order to improve the coil focusing performance, based on the addition of a magnetic core to the existing coils to enhance the stimulus intensity and focality of the local space. They designed the optimal configuration of the C-type core coil with a trade-off between stimulation effect and heat energy. Compared with the traditional Figure-of-Eight (FOE) coil, the focality of the C-type thin core coil is increased by 20.4–31.0%, and the maximum induced electric field intensity is increased by 44.7–67.3%.

With the aim of enhancing the overall performance of TMS treatments, Li et al. [66] and Zhang et al. [67] proposed a combination of two hardware systems, based on different physical concepts, i.e., ultrasound combined to RF magnetic coils. In more detail, in [66], the system is composed of an MRgFUS neuromodulation system RF coil and an MRI-compatible ultrasound device. In particular, the brain stimulation is performed by ultrasound beams, guided by MRI imaging. The developed MRI RF coil is designed to cover the whole considered brain to acquire MRI image; they compared the described system with a single loop, and they observed a 50% increase of SNR in the acquired MRI image resolution, also reducing the distortion. In modality “FUSon” (focused ultrasound turned on), ultrasound beams are used to excite brain tissue. The obtained results support the hybrid hardware approach to improve the entire system efficiency.

**Figure 5:**
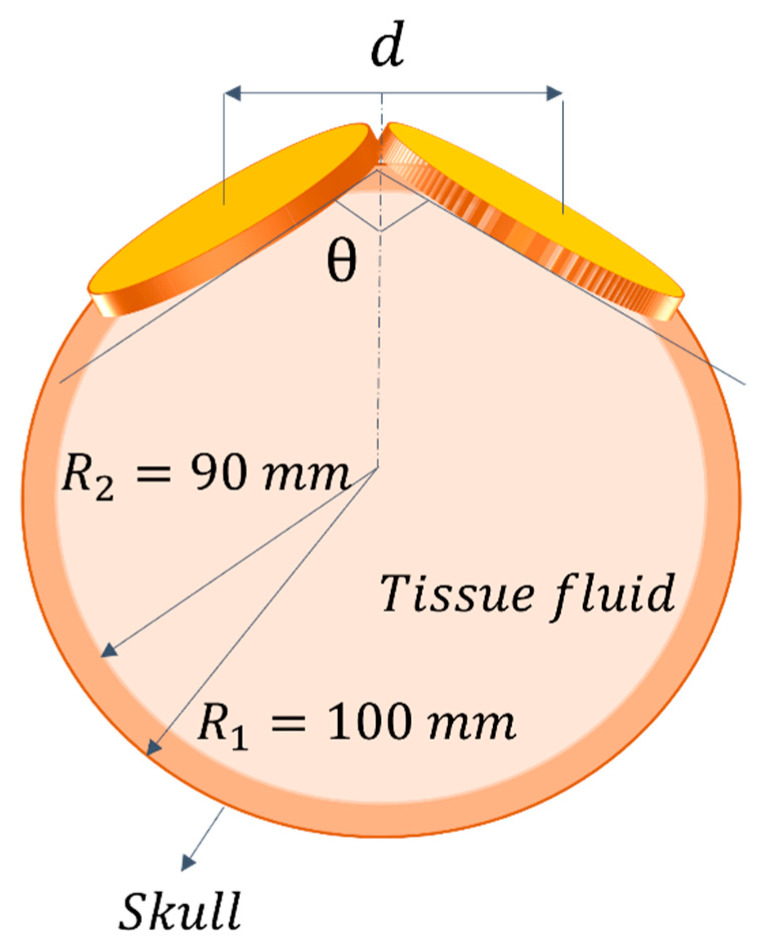
Biconical stimulator coils system: pictorial representation.

As introduced, as the targeted region depth increases, the magnetic field becomes less focused, and the stimulation effectiveness decreases. This is due to the magnetic field behavior, which is diffractive in nature. To cope with this issue, in [68], a biconical stimulation coil system is implemented, by using a finite-element method. They simulated a two-layer spherical human head model (Figure 5), representing the skull with the beneath brain tissue. This anatomical model, although simplified, allows them to accurately investigate the magnetic and electrical fields’ actual distributions. The authors observed that the magnetic field focalization and the stimulation depth, within the brain tissue, are mainly influenced by the two cones’ distance and the intersection angle. In particular, results showed that the magnetic field is more focused, and the stimulation depth increases when the distance between the two cones decreases (and the intersection angle correspondingly increases). This solution does not require complex instrumentation and it represents a feasible and effective TMS configuration.

In addition, another technique to deflect and deliver in the desired spatial region of the RF magnetic field is the adoption of magnetic shields. The effect and the contribution of a magnetic shield were studied by Meng Q. et al. [69]. In particular, they demonstrated the use of the magnetic shield to accomplish a significant magnetic field focusing by preserving, at the same time, the therapeutical throughput. The designed shield is composed of multiple layers of copper ring arrays, which utilize induced current to generate counter magnetic fields. In particular, the results showed how the focusing effect highly depends on the geometric design of the shield. The same innovative solution was also evaluated by Rastogi P. et al. [70], where the authors added a passive magnetic shield, consisting of a high-permeability ferromagnetic material, to a quadruple butterfly coil (QBC). The proposed configuration is able to improve the system performance in terms of focusing. In particular, they implemented two different solutions: a single and a double magnetic shield, evaluating also various positions within the brain. The obtained results showed an improvement in focality of 25% in both single and double shields cases with respect to the simple butterfly coil. These results support magnetic shield use to focus the magnetic field in the investigated tissue, highlighting that the use of the double shield, which increases the global system complexity, does not improve the results in terms of magnetic field focalization.

Another innovative approach to focusing the magnetic field is the implementation of an algorithm able to find the optimal hardware system configuration. Xiong H. et al. [71] proposed a solution based on magnetic field focusing through a hybrid algorithm based on a simplified particle swarm optimization algorithm (sPSO) and a simulating annealing (SA) algorithm. The simulation results showed that the hybrid algorithm and the system parameters optimalization can significantly improve the system focusing performance.

**Figure 6:**
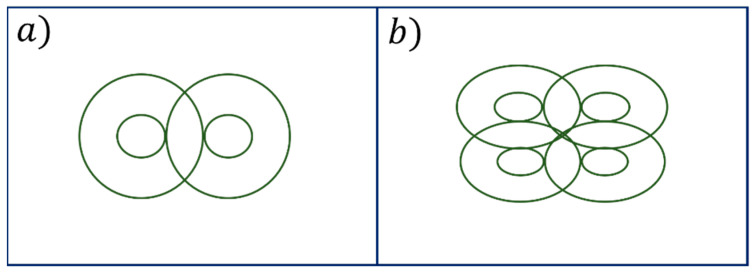
Perspective view of TMS figure-of-eight enhanced coils configurations (**a**), and the “four leaf solution” (**b**) [72].

Other authors, by starting from traditional radiating configurations, have introduced some modifications to improve performance. In particular, to enhance the figure of eight coil focusing capabilities, the classical configuration was designed with a ferromagnetic core, considering the oblique loop position which can cope with the irregular shape of the human scalp (Figure 6a). In particular, due to the magnetic field attenuation, the conformal hardware system design is an innovative solution to reduce the interface between the system and the human scalp. The distance between two loops was optimized to improve the electromagnetic field focusing and increase the stimulation depth. For the more specific purpose to enhance the strength of magnetic flux, they also implemented a four-leaf coil solution [72] (Figure 6b).

Following this path, an innovative configuration, similar to a “Halo coil”, was studied by March S.D. et al. [73]. In particular, the coil was designed by adopting three different technical constraints. Furthermore, the coil must generate a focused electric field around 150 V/m inside the investigated brain region; secondly, the magnetic flux at the surface of the coil must have values near 3 T; finally, the coil must support at least 5000 A current pulses at 2.5 kHz. The best-analyzed configuration, in terms of generated focused and strongest electric field, is composed of a combination of horizontal and vertical coils: in particular, the ten horizontal coils were placed perpendicularly to the smaller vertical coil.

Moreover, Shuo Yang, Guizhi, et al. [74] proposed an additional solution comparing, in terms of magnetic field focalization and eddy currents distribution, three hardware configurations: circular, figure-of-eight and circular coil array models. In particular, they observed that the stimulation intensity of a circular coil is less than the figure-of-eight coil. However, the field attenuation for the figure-of-eight coil is significantly higher. Therefore, the best results have been obtained with the circular coil array, where the stimulation intensity is maximized while the field attenuation is minimized with respect to the other two solutions.

**Figure 7:**
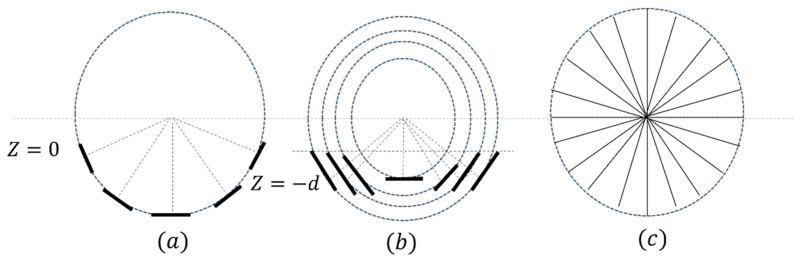
Different coil arrays configurations proposed in [75]: (**a**) hemispherical; (**b**) plane and (**c**) torus array.

Liu et al. [75], in their work, proposed an innovative scheme of coil arrays in hemispherical, plane and torus shapes to achieve a magnetic field distribution with ideal focusing capability (Figure 7). To evaluate the different coil arrays’ performance, they compared them in terms of magnetic and electric field focalization. A Self-Reparative and Adaptive Genetic Algorithm (SRAGA) is applied in the optimization of the currents infused into each coil.

To improve the magnetic nerve stimulation, Al-Mutawaly N. et al. [76] worked on an effective method to optimize magnetic field focusing and penetration depth. In fact, they proposed two new coil designs that can be used for magnetic stimulation of the peripheral or central nervous system. The two proposed designs are based on the use of a magnetic core coil and an air-core coil. They compared these designs with a figure-of-eight coil, in terms of magnetic field focalization. In particular, the obtained results showed the improvement of the system performance with the introduction of the ferromagnetic material.

Clearly, more complex configurations have also been developed. In the work by Xiong et al. [77], for instance, a new design composed of a three layers multi-coil (TLMC) is proposed. The designed structure significantly improves the focusing capability and stimulation strength, but it reduces the stimulation depth. To overcome this limitation, the authors introduced a particle swarm optimizer able to optimize the current configuration and the rotation angle of the fabricated TLMC.

In their study, instead, Zhang and Edrich [78], explored the use of *n*-plane or orthogonal combinations of eccentric coils to produce a desirable focusing spot. To avoid undesirable overstimulation near the healthy tissue, they generated opposite coil currents to produce field cancellation. This approach is very significant in terms of magnetic field shaping. In fact, it is possible to produce the desired hot spot by generating the appropriate current distribution.

In particular, the concept of temporal interference (TI) was introduced in [79], in order to achieve focal and steerable stimulation in the targeted brain area through transcranial magnetic stimulation. Furthermore, their solution was based on the design of two independent coils able to induce two high-frequency electric fields. This method works by exploiting the low-pass filter and the brain’s intrinsic nonlinear nature: the membrane does not allow the nerve to engage at high frequencies, unlike the neurons. Therefore, by optimizing the coils’ relative position and orientation, they controlled the intersection of the produced fields to have a deep stimulation and better spatial spread. Finally, changing the stimuli voltage ratios could make it possible to spatially steer the stimulation location without mechanical coil movements.

**Figure 8:**
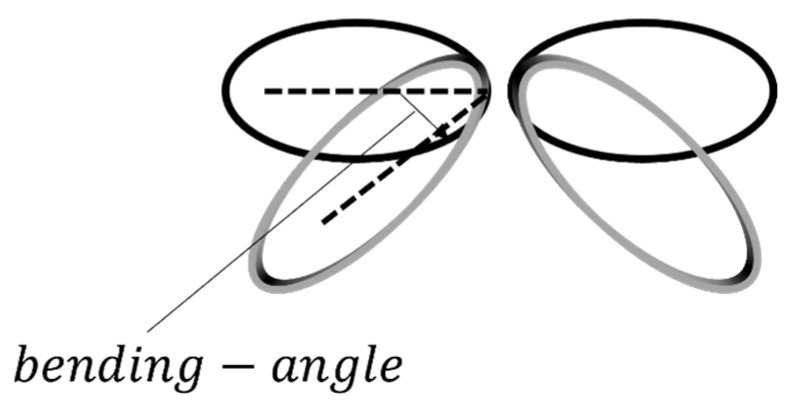
Bending-angle of the figure-of-eight coil evaluated in [80].

Tsuyama S. et al. [80] investigated the relationship between the induced eddy currents density and the coil configuration for effective stimulation. They evaluated the coil parameters such as the coil radius and the bending angle to stimulate a specific area for special figure-of-eight coils (Figure 8). In particular, they analyzed the eddy current distribution and value for different coil configurations and parameters. The obtained results showed that the maximum value of the eddy current and, consequently, the stimulated area, increases linearly with the increase in the coil radius and bending angle.

Finally, Bessel beams have been recently proposed also for TMS treatments. They consist of particular solutions to Maxwell’s equations, particularly useful in biomedical applications thanks to the peculiar nature to not suffer diffractive propagation and remain coherent with increasing distances. Rotundo S. et al. [81] proposed this physical concept by implementing an innovative radiating system for TMS. Moreover, the hardware solution is composed of five concentric planar spiral coils, working at typical TMS frequency, singularly and appropriately fed with proper current amplitude and phase. In this way, the Bessel-like non-diffractive magnetic field distribution, able to induce a focused electric field inside the investigated tissue, has been demonstrated. Compared to the single spiral coil, the obtained results showed that the Bessel Beam launcher presents a 48% smaller focal spot at 8 mm of depth.

To conclude, it is generally evident that, for TMS treatments, the major approach to focusing the magnetic field is the optimization of the hardware system parameters because of the strict correlation between the generated currents and the induced magnetic field distribution.

### 3.3. The Frontiers of Magnetic Field Shaping: Use of Metamaterials

**Figure 9:**
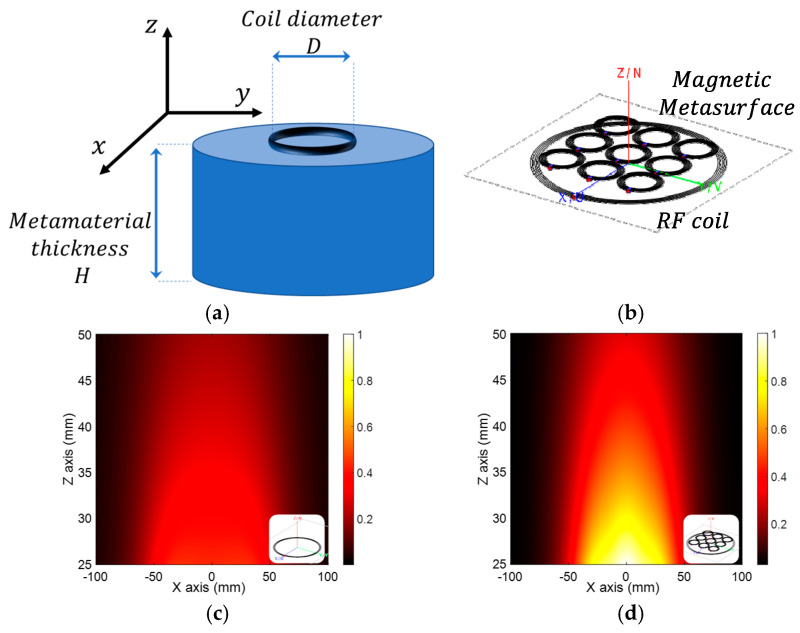
An ideal metamaterial slab placed in proximity of an active RF coil (**a**) can be practically realized through an opportunely designed array of resonating spiral resonators (**b**). For the same circulating current amplitude in the active RF coil, the metasurface presence is able to significantly enhance the produced magnetic field, facilitating the penetration inside biological tissues (**c**,**d**).

Recently, a huge research interest among the scientific community has been devoted to metamaterials and metasurfaces, due to the exotic and unnatural electromagnetic properties that can be achieved with similar solutions [82]. In fact, these promising artificial materials, properly designed, are able to generate not only negative dielectric permittivity or magnetic permeability but also negative refractive index. Such extraordinary properties are related to the intrinsic structure of metamaterials and metasurfaces, i.e., 3D and 2D matrices of resonating unit-cells whose dimensions are significantly smaller with respect to the applied wavelength. In this way, the impinging electromagnetic field is not able to distinguish the elementary structure, interpreting the metamaterial as a homogeneous and bulk material. Therefore, it is not surprising that metamaterials and metasurfaces have been also investigated in order to control specifically the magnetic field behavior, acting as filtering or focusing elements. In this sense, an array of split rings or spiral resonators are the most suited unit-cells to interact with the RF magnetic field (Figure 9a,b).

For these reasons, several solutions to focus the magnetic field at very low frequencies (a few MHz) have been proposed. To reach the desired field distribution synthesis, active metasurfaces have been developed. As the nomenclature suggests, in this solution each unit-cells can be independently fed in terms of current amplitude and phase [83,84]. In addition, a similar solution but exploiting passive metasurfaces has been described in [85]. In this work, a response-controlled passive magnetic metasurface acts as a filter of the magnetic field distribution generated by an active RF coil, achieving ultra-high focusing capability. In particular, the possibility of rapidly designing and configuring a passive metasurface allows us to obtain the desired magnetic field distribution, almost in an arbitrary way [86,87]. The described metasurface consisted of a 5 × 5 matrix of single loops, each having a 3 cm diameter and placed 1 cm apart from the others. Overall, the metasurface presented a dimension of 19 cm × 19 cm, and it was positioned 5 mm above the exciting RF coil. The numerical results showed the possibility to present a focal spot of 3 cm in diameter at a plane placed 8 cm above the metasurface, encouraging and validating the design methodology.

Additionally, Shuai et al. [88] explore the use of the metamaterials to reach a desired field distribution. In particular, negative refractive index metamaterials (MM) can be used to focus magnetic fields in the near-field. They implemented a quasi-static finite difference code to simulate eddy-currents inside the conductive brain when excited by magnetic fields focused through a MM lens, demonstrating that these systems in combination produce more compact brain excitations than lens-free setups.

Due to these materials’ versatility, magnetic metasurfaces have been also adopted in the MRI technique. Indeed, properly designed metasurfaces are able to facilitate the penetration of the RF magnetic field inside the human body, therefore leading to a significant SNR enhancement compared to traditional RF surface coils (Figure 9c,d).

In [89], the authors proposed a metasurface based on the 2D periodic arrangement of an innovative unit-cell, designed following the Hilbert parametric curves for a 7T MRI scanner.. This solution greatly enhanced the magnetic field penetration within tissues, allowing an up to 40% SNR enhancement. Similarly, a metasurface made by capacitively-loaded dipoles has been developed in [90], obtaining the same order of SNR enhancement.

One of these metasurfaces’ relevant problems is their capability to survive the high-power transmit pulse typical of an MRI scanner. Therefore, the work described in [91] introduced a passive but tunable metasurface to enhance the SNR and the sensitivity of the surface RF coil for the receiving phase whereas being out-of-frequency during the high power pulse produced by a volume birdcage coil working at the 3T Larmor frequency. The basic principle is to couple the metasurface—in this case, made by 2D arrangements of loaded dipoles—to an external tuning loop loaded with a varactor. Moreover, when the power impinging on this structure is high, the varactor saturates, changing its capacitance value and detuning the whole array, while the varactor retains the correct capacitance value when the system is used at the receiving phase low power, tuning the metasurface at the correct frequency.

Another approach is to recur to special active metasurfaces, in which the elementary unit-cells can be tuned on demand, able to reconfigure themselves to achieve optimized performance independent of the human district to be investigated. Following this principle, a metasurface whose unit-cells’ metal content can be changed by pumping liquid Hg in and out from the metasurface has been presented in [92]. Although this solution can be used to dynamically change the metasurface properties, nevertheless it is required a quite complicated system to allow the flux of Hg metal. A more convenient way to dynamically change the behavior of the metasurface consists in driving capacitive diodes loading the unit-cells with an external digital circuit [93], operating for a 1.5 T scanner. In this way, the metasurface can be in principle reconfigured even in a wireless way from the outside during an MRI scan.

### 3.4. Critical Comparison between the Different Radiating Solutions

By reporting three exemplificative biomedical applications, we have reviewed several radiating solutions appeared so far in the literature to manipulate the magnetic field distribution. In particular, volume inductors are probably the simplest configuration to illuminate large regions. Nevertheless, substantial differences can be highlighted between solenoids, Helmholtz and birdcage coils. Indeed, solenoids produce a quite homogeneous magnetic field distribution within their volume, provided that their length is significantly greater than the sectional diameter; moreover, they are flexible and scalable in terms of geometry. Instead, Helmholtz coils are able to radiate a very homogeneous magnetic field distribution, but consistently high voltages are required; in other words, this design is less efficient than solenoids. Finally, Birdcage coils are also widely diffused because they can generate a homogeneous linear or circularly polarized magnetic field; their straightforward compatibility with MRI makes them extremely useful in imaging-guided hyperthermic treatments.

Clearly, volume coils are not suited when a confined and targeted illumination must be provided, as in ablation or TMS for instance. In this case, surface coils demonstrated to be the most promising solution to stimulate small and more superficial regions with high efficiency. For example, as a rule of thumb, for maximizing the sensitivity in the FOV, the coil radius for a circular surface loop should roughly match the desired imaging depth [35]. Moreover, since their dimensions are reduced, surface coils produce less undesired eddy currents, confining the treatment. Precisely, surface coils are able to maximize the Power Ratio (PR) with respect to volume coils for short distances in magnetic hyperthermic treatments. As explained, PR is defined as the ratio between the induced heat in MNPs and the undesired surface eddy current heating. Moreover, they can be more easily tuned and matched with respect to the generator, therefore requiring lower reactive power. Particular designs, such as the pancake, can also achieve extremely high levels of miniaturization, guaranteeing very accurate stimulations. It is well known that, in general, surface coils need a dedicated cooling system (for instance, with flowing water) or Litz wires to dissipate power; this is due to the difficulties to eliminate heat in their very nested and small structure.

Transcranial Magnetic Stimulation, because of the strict requirement to confine the magnetic field so as to not excite undesired portions of brain tissue, allows exploring other technical solutions. Ferromagnetic slabs or field concentrators are excellent candidates to increase the shaping performance, directing the magnetic field towards the desired target. In addition, it has been also proposed to adopt magnetic shields based on induced currents on copper rings, able to cancel the undesired radiation. Other promising and effective radiating systems are constituted by variations of the classical figure-of-eight coil and by non-diffractive Bessel-beam launchers. The latter configurations are able to precisely excite a defined tissue target due to the collimated beam. Lastly, array arrangements have been described to enhance the performance of the single radiating element; in this sense, optimization algorithms have been proposed to retrieve the best array design.

Finally, metamaterials and metasurfaces for magnetic field distribution manipulation have been reviewed. It has been shown the great potentiality of similar solutions thanks to which an unprecedented capability of field control can be achieved. For this reason, the number of applications, especially in the biomedical field, is dramatically increasing, both for passive and active solutions. In particular, while the passive configurations are able to deal with high-power applications, nevertheless the active ones allow reconfigurability which can be significantly important for smart solutions.

A comprehensive summary of the various radiating solutions has been reported in Table 1.

## 4. Conclusions

In recent years, the use of RF magnetic fields in biomedical applications, for both diagnosis and therapy, has dramatically increased. In the last decades, MRI, a non-invasive medical imaging technique for diagnostic purposes, has met a widespread application in clinical settings. In parallel, techniques exploiting RF magnetic fields for local treatment of different pathologies have recently emerged. Shaping and controlling the magnetic field distribution is one of the most important issues for biomedical techniques exploiting RF magnetic fields, both for optimizing treatment and diagnosis efficacy and for ensuring the safety of the operative procedures. Because of the different technologies’ purposes, the desired magnetic field shaping is dependent on the considered applications; in particular, it is necessary to produce a homogeneous magnetic field to improve the diagnostic FOV in MRI, while it is desirable to focus the field distribution in treatment technologies, as MHF and TMS.

MFH is a promising cancer treatment technique that consists in inducing a temperature increase in magnetic particles’ fluids (injected directly into the tumor tissue or functionalized in order to reach the tumor after systemic administration), by means of an alternating RF magnetic field. The ability to provide highly localized heat deposition without damaging the healthy cells surrounding the tumor is one of the main advantages of this technique. The hyperthermia treatment effectiveness is strictly dependent on the chemophysical properties of the magnetic particles, as well as on their ability to reach the target tissue. However, the development of an optimized setup for RF magnetic field generation and focusing on the target volume is of utmost importance, since the lack of focus could result in great exposure of healthy tissues, as well as in failure to achieve a uniform temperature increase and to comply with safety conditions. In this review paper, we report an overview of the main inductors’ designs proposed in the literature for optimal focusing of RF magnetic field in hyperthermia studies. As previously described, inductors can be classified by volume (such as the solenoid or the Helmholtz) or surface (such as the pancake) configurations, and the choice of the best-suited design is strictly related to the specific experimental model, as well as to the location and dimensions of the target region. The main problem associated with the use of volume inductors is the need to achieve high magnetic field homogeneity. Several technical solutions, such as the use of magnetic field concentrators, rings or the adoption of geometrical modifications, have been investigated for this purpose. Surface inductors provide a higher magnetic field intensity that rapidly falls off with distance from the coil, thus producing a smaller region of the usable magnetic field. Similar technological setups have been also proposed for magnetic ablation studies in which tissue heating is exploited to induce extensive tumor cell necrosis.

Instead, TMS is a non-invasive electromagnetic technique for brain stimulation, applied in the clinical treatment and in the study of functional activities in Parkinson’s disease and in neuronal and psychiatric disorders, such as depression. TMS works in a contactless way; it is painless, less expensive, faster, and, at least in some cases, more effective than conventional electrical stimulation. Typically, the hardware system is located near the scalp in order to generate a magnetic field that, in turn, induces an electric field and eddy currents in the conductive brain tissue. When the spatial gradient of the induced electric field aligns with a nerve fiber, an action potential is generated. Nevertheless, TMS coils generate rather diffused fields that decay rapidly and diffract with the increasing distance from the source. These aspects often lead to the rise of fields also exciting the surrounding tissues around the investigated target region and/or fail to reach it entirely. For these reasons, it would be highly necessary to optimize the generated field focalization, and consequently, the induced electric field spot, at different depths, to accurately and efficiently excite the target. Due to the brain complexity and the natural magnetic field propagation, generating a desired magnetic, and consequently, an induced electric field spot is technically challenging. Several technological solutions have been proposed so far to overcome these limitations: the approaches reported in the literature involve both optimized hardware system development (for instance by using ultrasound stimulation or by modifying the design and geometry of the TMS coil loops), and optimized simulation algorithms. An overview of the most promising solutions has been discussed.

Finally, recent advances in the context of magnetic field focusing and shaping have been developed through the use of electromagnetic metamaterials and metasurfaces. Due to their intrinsic structure, they provide negative dielectric permittivity, magnetic permeability and refractive index, showing unnatural electromagnetic properties. As reported in this review paper, several solutions (relating not only to MRI) rely on metamaterials and metasurfaces to tailor the magnetic field distribution and to achieve ultra-high focusing capability.

In conclusion, the development of novel and optimized approaches for RF magnetic field focusing is of primary importance in various biomedical applications, such as magnetic hyperthermia and ablation, transcranial magnetic stimulation and magnetic resonance imaging. Some of the recent advances in this field have been described in this review. This review paper could be useful to fill the actual gap in the literature and to serve as a guide for the physicians and engineers working in the context of biomedical and bioengineering techniques exploiting magnetic fields.

## Figures and Tables

**Table 1:** Comparison between the radiating system able to shape and control the magnetic field distribution.

**Volume Coils**	**Pros**	**Cons**	**Refs.**
*Solenoid*	Medium homogeneity Geometrically scalable High efficiency	The length must be greater than section	[42,48,49,50,51,54,55]
*Helmholtz*	Very homogeneous field	Poor efficiency	[43,44,46]
*Birdcage*	Homogeneous circularly or linearly polarized field	Design complexity	[22,57]
**Surface Coils**	**Pros**	**Cons**	**Refs.**
*Pancake*	Miniaturized Reduced undesired exposition Possibility to adopt shield and magnetic cores to increase performance	High temperature for high-power operation: cooling system Superficial treatment	[41,45,60]
*Figure-of-eight*	Geometrically re-tunable (angle, distance) Reduced undesired exposition Possibility to adopt shields and magnetic cores to increase performance	High temperature for high-power operation: cooling system Cumbersome	[65,69,70,72,74,76]
*Bessel-beam*	Non diffractive magnetic field beam Accurate stimulation	Very rapid magnetic field amplitude decay with distances	[81]
*Arrays*	Enhanced performance with respect to a single coil Use of optimization algorithm to create array with enhanced performance	More complex design Cumbersome	[75,77,78]
**Metamaterials and** **Metasurfaces**	**Pros**	**Cons**	**Refs.**
*Passive* *metasurface*	High level of field distribution control by filtering action Able to sustain high power operation	Not reconfigurable	[85,86,87,90,91]
*Active* *metasurface*	High level of field distribution control by filtering action Reconfigurable	Electronic elements sensitive to high power pulses	[83,84,92,93]

## Data Availability

Not applicable.

## References

[1] Obaidat I.M., Narayanaswamy V., Alaabed S., Sambasivam S., Muralee Gopi C.V. (2019). Principles of magnetic hyperthermia: A focus on using multifunctional hybrid magnetic nanoparticles. Magnetochemistry.

[2] Etlï E.E., Akar A. (2022). Magnetic nanoparticles for diagnosis and treatment. Medicine.

[3] Rosensweig R.E. (2002). Heating magnetic fluid with alternating magnetic field. J. Magn. Magn. Mater..

[4] Gilchrist R.K., Medal R., Shorey W.D., Hanselman R.C., Parrott J.C., Taylor C.B. (1957). Selective inductive heating of lymph nodes. Ann. Surg..

[5] Hilger I. (2013). In vivo applications of magnetic nanoparticle hyperthermia. Int. J. Hyperth..

[6] Piao D., Le K., Saunders D., Smith N., Goddard J., Figueroa D., Krasinski J.S., Chen W.R., Towner R.A. Development of a vertically and horizontally applicable multi-frequency alternating-magnetic-field device for hyperthermia of glioma in rodent model using iron oxide based nanoparticles. Proceedings of the Biomedical Optics Optical Society of America.

[7] Dutz S., Hergt R. (2013). Magnetic nanoparticle heating and heat transfer on a microscale: Basic principles, realities and physical limitations of hyperthermia for tumour therapy. Int. J. Hyperth..

[8] Zhao Q., Wang L., Cheng R., Mao L., Arnold R.D., Howerth E.W., Chen Z.G., Platt S. (2012). Magnetic nanoparticle-based hyperthermia for head & neck cancer in mouse models. Theranostics.

[9] Kumar C.S., Mohammad F. (2011). Magnetic nanomaterials for hyperthermia-based therapy and controlled drug delivery. Adv. Drug Deliv. Rev..

[10] Stapf M., Pömpner N., Kettering M., Hilger I. (2015). Magnetic thermoablation stimuli alter BCL2 and FGF-R1 but not HSP70 expression profiles in BT474 breast tumors. Int. J. Nanomed..

[11] Skumiel A., Leszczyński B., Molcan M., Timko M. (2016). The comparison of magnetic circuits used in magnetic hyperthermia. J. Magn. Magn. Mater..

[12] Lu Y., Rivera-Rodriguez A., Tay Z.W., Hensley D., Fung K.B., Colson C., Saayujya C., Huynh Q., Kabuli L., Fellows B. (2020). Combining magnetic particle imaging and magnetic fluid hyperthermia for localized and image-guided treatment. Int. J. Hyperth..

[13] Pucci C., Degl’Innocenti A., Gümüş M.B., Ciofani G. (2022). Superparamagnetic iron oxide nanoparticles for magnetic hyperthermia: Recent advancements, molecular effects, and future directions in the omics era. Biomater. Sci..

[14] Rodrigues H.F., Capistrano G., Bakuzis A.F. (2020). In vivo magnetic nanoparticle hyperthermia: A review on preclinical studies, low-field nano-heaters, noninvasive thermometry and computer simulations for treatment planning. Int. J. Hyperth..

[15] Laurent S., Dutz S., Häfeli U.O., Mahmoudi M. (2011). Magnetic fluid hyperthermia: Focus on superparamagnetic iron oxide nanoparticles. Adv. Colloid Interface Sci..

[16] Kaur P., Aliru M.L., Chadha A.S., Asea A., Krishnan S. (2016). Hyperthermia using nanoparticles–promises and pitfalls. Int. J. Hyperth..

[17] Jiao W., Zhang T., Peng M., Yi J., He Y., Fan H. (2022). Design of Magnetic Nanoplatforms for Cancer Theranostics. Biosensors.

[18] Connord V., Mehdaoui B., Tan R.P., Carrey J., Respaud M. (2014). An air-cooled Litz wire coil for measuring the high frequency hysteresis loops of magnetic samples—A useful setup for magnetic hyperthermia applications. Rev. Sci. Instrum..

[19] Garaio E., Collantes J.M., Plazaola F., Garcia J.A., Castellanos-Rubio I. (2014). A multifrequency eletromagnetic applicator with an integrated AC magnetometer for magnetic hyperthermia experiments. Meas. Sci. Technol..

[20] Tasci T.O., Vargel I., Arat A., Guzel E., Korkusuz P., Atalar E. (2009). Focused RF hyperthermia using magnetic fluids. Med. Phys..

[21] Iszály Z., Márián I.G., Szabó I.A., Trombettoni A., Nándori I. (2022). Theory of superlocalized magnetic nanoparticle hyperthermia: Rotating versus oscillating fields. J. Magn. Magn. Mater..

[22] Gresits I., Thuróczy G., Sági O., Gyüre-Garami B., Márkus B.G., Simon F. (2018). Non-calorimetric determination of absorbed power during magnetic nanoparticle based hyperthermia. Sci. Rep..

[23] Abu-Bakr A.F., Zubarev A.Y. (2019). Hyperthermia in a system of interacting ferromagnetic particles under rotating magnetic field. J. Magn. Magn. Mater..

[24] Konopacki M., Jędrzejczak-Silicka M., Szymańska K., Mijowska E., Rakoczy R. (2021). Effect of rotating magnetic field on ferromagnetic structures used in hyperthermia. J. Magn. Magn. Mater..

[25] Beković M., Trlep M., Jesenik M., Hamler A. (2014). A comparison of the heating effect of magnetic fluid between the alternating and rotating magnetic field. J. Magn. Magn. Mater..

[26] Ivkov R., DeNardo S.J., Daum W., Foreman A.R., Goldstein R.C., Nemkov V.S., DeNardo G.L. (2005). Application of high amplitude alternating magnetic fields for heat induction of nanoparticles localized in cancer. Clin. Cancer Res..

[27] Ashikbayeva Z., Tosi D., Balmassov D., Schena E., Saccomandi P., Inglezakis V. (2019). Application of nanoparticles and nanomaterials in thermal ablation therapy of cancer. Nanomaterials.

[28] Hilger I., Hiergeist R., Hergt R., Winnefeld K., Schubert H., Kaiser W.A. (2002). Thermal ablation of tumors using magnetic nanoparticles: An in vivo feasibility study. Investig. Radiol..

[29] Johannsen M., Thiesen B., Wust P., Jordan A. (2010). Magnetic nanoparticle hyperthermia for prostate cancer. Int. J. Hyperth..

[30] Bredlau A.-L., McCrackin M.A., Motamarry A., Helke K., Chen C., Broome A.-M., Haemmerich D. (2016). Thermal therapy approaches for treatment of brain tumors in animals and humans. Crit. Rev. Biomed. Eng..

[31] Bruners P., Braunschweig T., Hodenius M., Pietsch H., Penzkofer T., Baumann M., Günther R.W., Schmitz-Rode T., Mahnken A.H. (2010). Thermoablation of malignant kidney tumors using magnetic nanoparticles: An in vivo feasibility study in a rabbit model. Cardiovasc. Interv. Radiol..

[32] Moore J., Xu S., Wood B.J., Ren H., Tse Z.T.H. (2020). Radiofrequency tumor ablation system with a wireless or implantable probe. Wirel. Power Transf..

[33] Matsui H., Hamuro M., Nakamura K., Kayahara H., Murano K., Kotsuka Y., Miki Y. (2012). Development of a highly efficient implanted thermal ablation device: In vivo experiment in rat liver. Br. J. Radiol..

[34] Jin J. (2018). Electromagnetic Analysis and Design in Magnetic Resonance Imaging.

[35] Haase A., Odoj F., Von Kienlin M., Warnking J., Fidler F., Weisser A., Nittka M., Rommel E., Lanz T., Kalusche B. (2000). NMR probeheads for in vivo applications. Concepts Magn. Reson..

[36] Roemer P.B., Edelstein W.A., Hayes C.E., Souza S.P., Mueller O.M. (1990). The NMR phased array. Magn. Reson. Med..

[37] Giovannetti G. (2016). Comparison between circular and square loops for low-frequency magnetic resonance applications: Theoretical performance estimation. Concepts Magn. Reson. Part B: Magn. Reson. Eng..

[38] Giovannetti G., Hartwig V., Positano V., Vanello N. (2014). Radiofrequency coils for magnetic resonance applications: Theory, design, and evaluation. Crit. Rev. Biomed. Eng..

[39] Collick B.D., Behzadnezhad B., Hurley S.A., Mathew N.K., Behdad N., Lindsay S.A., Robb F., Stormont R.S., McMillan A.B. (2020). Rapid development of application-specific flexible MRI receive coils. Phys. Med. Biol..

[40] Zamarayeva A.M., Gopalan K., Corea J.R., Liu M.Z., Pang K., Lustig M., Arias A.C. (2021). Custom, spray coated receive coils for magnetic resonance imaging. Sci. Rep..

[41] Brizi D., Fontana N., Giovannetti G., Menichetti L., Cappiello L., Doumett S., Ravagli C., Baldi G., Monorchio A. (2019). A Radiating System for Low-Frequency Highly Focused Hyperthermia with Magnetic Nanoparticles. IEEE J. Electromagn. RF Microw. Med. Biol..

[42] Stauffer P.R., Sneed P.K., Hashemi H., Phillips T.L. (1994). Practical induction heating coil designs for clinical hyperthermia with ferromagnetic implants. IEEE Trans. Biomed. Eng..

[43] Nemkov V., Ruffini R., Goldstein R., Jackowski J., DeWeese T.L., Ivkov R. (2011). Magnetic field generating inductor for cancer hyperthermia research. COMPEL-Int. J. Comput. Math. Electr. Electron. Eng..

[44] Nieskoski M.D., Trembly B.S. (2014). Comparison of a single optimized coil and a Helmholtz pair for magnetic nanoparticle hyperthermia. IEEE Trans. Biomed. Eng..

[45] Blanco-Andujar C., Ortega D., Southern P., Nesbitt S.A., Thanh N.T.K., Pankhurst Q.A. (2016). Real-time tracking of delayed-onset cellular apoptosis induced by intracellular magnetic hyperthermia. Nanomedicine.

[46] Hadadian Y., Azimbagirad M., Navas E.A., Pavan T.Z. (2019). A versatile induction heating system for magnetic hyperthermia studies under different experimental conditions. Rev. Sci. Instrum..

[47] Tang Y., Jin T., Flesch R.C., Gao Y. (2020). Improvement of solenoid magnetic field and its influence on therapeutic effect during magnetic hyperthermia. J. Phys. D Appl. Phys..

[48] Cano M.E., Barrera A., Estrada J.C., Hernandez A., Cordova T. (2011). An induction heater device for studies of magnetic hyperthermia and specific absorption ratio measurements. Rev. Sci. Instrum..

[49] Mazon E.E., Sámano A.H., Calleja H., Quintero L.H., Paz J.A., Cano M.E. (2017). A frequency tuner for resonant inverters suitable for magnetic hyperthermia applications. Meas. Sci. Technol..

[50] Brizi D., Fontana N., Giovannetti G., Flori A., Menichetti L., Doumett S., Baldi G., Monorchio A. (2018). A novel approach for determining the electromagnetic properties of a colloidal fluid with magnetic nanoparticles for hyperthermia applications. IEEE J. Electromagn. RF Microw. Med. Biol..

[51] Di Barba P., Dughiero F., Sieni E. (2010). Magnetic field synthesis in the design of inductors for magnetic fluid hyperthermia. IEEE Trans. Magn..

[52] Bertani R., Ceretta F., Di Barba P., Dughiero F., Forzan M., Michelin R.A., Sgarbossa P., Sieni E., Spizzo F. (2015). Optimal inductor design for nanofluid heating characterisation. Eng. Comput..

[53] Subramanian M., Miaskowski A., Pearce G., Dobson J. (2016). A coil system for real-time magnetic fluid hyperthermia microscopy studies. Int. J. Hyperth..

[54] Frijia F., Flori A., Giovannetti G. (2021). Design, simulation, and test of surface and volume radio frequency coils for 13C magnetic resonance imaging and spectroscopy. Rev. Sci. Instrum..

[55] Bordelon D.E., Goldstein R.C., Nemkov V.S., Kumar A., Jackowski J.K., DeWeese T.L., Ivkov R. (2011). Modified solenoid coil that efficiently produces high amplitude AC magnetic fields with enhanced uniformity for biomedical applications. IEEE Trans. Magn..

[56] Attaluri A., Jackowski J., Sharma A., Kandala S.K., Nemkov V., Yakey C., DeWeese T.L., Kumar A., Goldstein R.C., Ivkov R. (2020). Design and construction of a Maxwell-type induction coil for magnetic nanoparticle hyperthermia. Int. J. Hyperth..

[57] Giovannetti G., Landini L., Santarelli M.F., Positano V. (2002). A fast and accurate simulator for the design of birdcage coils in MRI. Magn. Reson. Mater. Phys. Biol. Med..

[58] Giovannetti G., Francesconi R., Landini L., Viti V., Santarelli M.F., Positano V., Benassi A. (2004). A quadrature lowpass birdcage coil for a vertical low field MRI scanner. Concepts Magn. Reson. Part B Magn. Reson. Eng. Educ. J..

[59] Wu Z., Zhuo Z., Cai D., Wu J., Wang J., Tang J. (2015). An induction heating device using planar coil with high amplitude alternating magnetic fields for magnetic hyperthermia. Technol. Health Care.

[60] Dürr S., Schmidt W., Janko C., Kraemer H.P., Tripal P., Eiermann F., Tietze R., Lyer S., Alexiou C. (2013). A novel magnetic field device for inducing hyperthermia using magnetic nanoparticles. Biomed. Eng. Biomed. Tech..

[61] Giovannetti G., Menichetti L. (2017). Litz wire RF coils for low frequency NMR applications. Measurement.

[62] Lacroix L.-M., Carrey J., Respaud M. (2008). A frequency-adjustable electromagnet for hyperthermia measurements on magnetic nanoparticles. Rev. Sci. Instrum..

[63] Nieminen J.O., Sinisalo H., Souza V.H., Malmi M., Yuryev M., Tervo A.E., Stenroos M., Milardovich D., Korhonen J.T., Koponen L.M. (2022). Multi-locus transcranial magnetic stimulation system for electronically targeted brain stimulation. Brain Stimul..

[64] Zhou H., Wang Y., Zhang W., Luo F., Han R. (2020). Simulation Research on Focusing Characteristics of 8-shaped Magnetic Coil. Proceedings of the 2020 IEEE International Conference on High Voltage Engineering and Application (ICHVE).

[65] Liu C., Ding H., Fang X., Wang Z. (2020). Optimal Design of Transcranial Magnetic Stimulation Thin Core Coil With Trade-Off Between Stimulation Effect and Heat Energy. IEEE Trans. Appl. Supercond..

[66] Li Y., Lee J., Long X., Qiao Y., Ma T., He Q., Cao P., Zhang X., Zheng H. (2020). A Magnetic Resonance-Guided Focused Ultrasound Neuromodulation System With a Whole Brain Coil Array for Nonhuman Primates at 3 T. IEEE Trans. Med. Imaging.

[67] Zhang S., Wang Z., Hou W., Zhao M., Xu G. Design of a new low-intensity focused ultrasound stimulation system with homogeneous magnetic field. Proceedings of the 2016 Asia-Pacific International Symposium on Electromagnetic Compatibility (APEMC).

[68] Wu Y.X., Yu H.Y., Liu Z.W. (2018). Numerical Investigation of the Magnetic and Electric Field Distributions Produced by Biconical Transcranial Magnetic Stimulation Coil for Optimal Design. IEEE Trans. Magn..

[69] Meng Q., Cherry M., Refai A., Du X., Lu H., Hong E., Yang Y., Choa F.-S. (2018). Development of Focused Transcranial Magnetic Stimulation for Rodents by Copper-Array Shields. IEEE Trans. Magn..

[70] Rastogi P., Tang Y., Zhang E., Lee G., Hadimani R.L., Jiles D.C. (2017). Quadruple Butterfly Coil With Passive Magnetic Shielding for Focused Transcranial Magnetic Stimulation. IEEE Trans. Magn..

[71] Xiong H., Shi J.H., Hu X.-W., Li J.-Z. (2016). The focusing Optimization of transcranial magnetic stimulation system. Prog. Electromagn. Res. M.

[72] Hasan M.M., Sufian S.M.A., Mehdi H., Siddique-e-Rabbani K. (2016). Designing a transcranial magnetic stimulator coil for Deep Brain Stimulation. Proceedings of the 2016 9th International Conference on Electrical and Computer Engineering (ICECE).

[73] March S.D., McAtee S., Senter M., Spoth K., Stiner D.R., Crowther L.J., Hadimani R.L., Jiles D.C. Focused and deep brain magnetic stimulation using new coil design in mice. Proceedings of the 2013 6th International IEEE/EMBS Conference on Neural Engineering (NER).

[74] Yang S., Xu G., Wang L., Geng Y., Yu H., Yang Q. (2010). Circular Coil Array Model for Tran-scranial Magnetic Stimulation. IEEE Trans. Appl. Supercond..

[75] Liu J., Lu J., Liu C., Hu Y. Coil Arrays Modeling and Optimization for Transcranial Magnetic Stimulation. Proceedings of the 2009 2nd International Conference on Biomedical Engineering and Informatics.

[76] Al-Mutawaly N., de Bruin H., Findlay D. Magnetic nerve stimulation: Field focality and depth of penetration. Proceedings of the 2001 Conference Annual International Conference of the IEEE Engineering in Medicine and Biology Society.

[77] Xiong H., Li Q., Liu J. (2021). Performance Optimization and Simulation Research of New Coil for Transcranial Magnetic Stimulation Based on Improved Particle Swarm Optimizer. IEEE Trans. Magn..

[78] Zhang T., Edrich J. Excentric coils for focused neuromagnetic stimulation and biomagnetic detection. Proceedings of 18th Annual International Conference of the IEEE Engineering in Medicine and Biology Society.

[79] Sorkhabi M.M., Wendt K., Denison T. Temporally Interfering TMS: Focal and Dynamic Stimulation Location. Proceedings of the 42nd Annual International Conference of the IEEE Engineering in Medicine & Biology Society (EMBC).

[80] Tsuyama S., Katayama Y., Hyodo A., Hayami T., Ueno S., Iramina K. (2009). Effects of Coil Parameters on the Stimulated Area by Transcranial Magnetic Stimulation. IEEE Trans. Magn..

[81] Rotundo S., Brizi D., Monorchio A. Bessel Beam Radiating System for Focused Transcranial Magnetic Stimulation. Proceedings of the 2022 16th European Conference on Antennas and Propagation (EuCAP).

[82] Pendry J.B., Holden A.J., Robbins D.J., Stewart W.J. (1999). Magnetism from conductors and enhanced nonlinear phenomena. IEEE Trans. Microw. Theory Tech..

[83] Choi B.H., Kim J.H., Cheon J.P., Rim C.T. (2016). Synthesized magnetic field focusing using a current-controlled coil array. IEEE Magn. Lett..

[84] Kim J.H., Choi B.H., Kim H.R., Rim C.T. (2019). 2-D Synthesized Magnetic Field Focusing Technology With Loop Coils Distributed in a Rectangular Formation. IEEE Trans. Ind. Electron..

[85] Falchi M., Rotundo S., Brizi D., Monorchio A. A Design Methodology for Response-controlled Passive Magnetic Metasurfaces. Proceedings of the 2022 16th European Conference on Antennas and Propagation (EuCAP).

[86] Brizi D., Monorchio A. (2021). An Analytical Approach for the Arbitrary Control of Magnetic Metasurfaces Frequency Response. IEEE Antennas Wirel. Propag. Lett..

[87] Brizi D., Monorchio A. (2022). Magnetic metasurfaces properties in the near field regions. Sci. Rep..

[88] Gomez L., Hernandez L., Grbic A., Michielssen E. A simulation of focal brain stimulation using metamaterial lenses. Proceedings of the 2010 IEEE Antennas and Propagation Society International Symposium.

[89] Motovilova E., Huang S.Y. (2019). Hilbert Curve-Based Metasurface to Enhance Sensitivity of Radio Frequency Coils for 7-T MRI. IEEE Trans. Microw. Theory Tech..

[90] Kretov E.I., Shchelokova A.V., Slobozhanyuk A.P. (2019). Control of the magnetic near-field pattern inside MRI machine with tunable metasurface. Appl. Phys. Lett..

[91] Stoja E., Konstandin S., Philipp D., Wilke R.N., Betancourt D., Bertuch T., Jenne J., Umathum R., Günther M. (2021). Improving magnetic resonance imaging with smart and thin metasurfaces. Sci. Rep..

[92] Wang H., Huang H.-K., Chen Y.-S., Zhao Y. (2021). On-demand field shaping for enhanced magnetic resonance imaging using an ultrathin reconfigurable metasurface. VIEW.

[93] Saha S., Pricci R., Koutsoupidou M., Cano-Garcia H., Katana D., Rana S., Kosmas P., Palikaras G., Webb A., Kallos E. (2020). A smart switching system to enable automatic tuning and detuning of metamaterial resonators in MRI scans. Sci. Rep..

